# Developmental trajectories of loneliness and social support in older adults: based on the parallel process latent growth curve model

**DOI:** 10.3389/fpubh.2025.1625322

**Published:** 2025-07-08

**Authors:** Chang Hu, Yuhan Chen, Xin Li, Xi Wang, Jin Chu

**Affiliations:** ^1^General Education Department, Wuhan College, Wuhan, China; ^2^School of Public Health Nursing, Hangzhou Normal University, Hangzhou, China; ^3^Department of Sociology, University of Manchester, Manchester, United Kingdom; ^4^School of Sociology, Nankai University, Tianjin, China

**Keywords:** social support, loneliness, cross-lagged model, parallel process latent growth curve model, older adults

## Abstract

**Background:**

With the acceleration of global aging, loneliness among older adults has become a prominent issue and a critical public health concern. Existing research has primarily focused on the cross-sectional relationship between social support and loneliness, but longitudinal dynamics and bidirectional mechanisms remain underexplored. This study aims to explore the developmental trajectories and interaction between loneliness and social support among Chinese older residents in the community.

**Methods:**

The study was conducted with three waves of data collection (6-month intervals) over 1 years, involving 1,225 Chinese older residents in the community. The Navigating the Social Support Scale and the UCLA Loneliness Scale were used to measure social support and loneliness, respectively. Cross-lagged panel modeling (CLPM) was employed to examine bidirectional predictive relationships, while parallel process latent growth curve modeling (LGCM) was applied to assess associations between initial levels and developmental rates of the two constructs.

**Results:**

(1) The loneliness of the older adults gradually increased over time, while the level of social support slowly decreased. (2) Loneliness could negatively predict social support from T1 to T2, and T2 to T3, but only social support at T1 negatively predicted loneliness at T2. (3) The initial level of loneliness could negatively predict the development speed of social support, and social support could also negatively predict the development speed of loneliness.

**Conclusion:**

We found that that high loneliness is a risk factor in the development of social support levels, and high social support is also a protective factor in the development of loneliness, which provides empirical evidence for the study of emotional health in the older adults.

## Introduction

Modern individuals are becoming increasingly lonely, with nearly one-third of people in industrialized nations found to suffer from loneliness (1). Loneliness has a profound effect on mental health (2). It is important to note that, with the acceleration of global aging, the mental health of the older adults has increasingly become a critical issue in public health (3). According to recent statistics, the older population over the age of 60 in China has surpassed 267 million, accounting for 18.9% of the total population (3). Alongside these profound demographic changes, the issue of loneliness among the older adults has garnered widespread attention from various sectors of society (4, 5). Epidemiological studies suggest that approximately 40% of the older population in China experiences varying degrees of loneliness, with 6.3% exhibiting severe symptoms (3, 6). Therefore, it is imperative to explore the factors behind loneliness. The emergence and persistence of loneliness among the older adults is shaped by multiple psychological and social factors (7, 8). According to socio-emotional selectivity theory (9, 10), as individuals age, their perception of time changes, which leads them to place greater emphasis on emotional fulfillment. This shift in priorities makes older adults more likely to seek close social relationships compared to younger individuals (7). That is, social support plays a key role in loneliness of the older adults. Previous studies have found that social support is a negative predictor of loneliness among the older adults (11). However, previous studies are cross-sectional designs and unable to test the long-term prospective association between social support and loneliness (11, 12). This reduces confidence in the ability to derive reliable causal inferences. Although a longitudinal study finds that social support of the older adults significantly predicts loneliness (13), such findings primarily focus on between-person differences and offer limited insight into intraindividual processes. That is, it remains unclear whether fluctuations in an individual’s perceived social support are associated with corresponding changes in their feelings of loneliness—a within-person association that is critical for understanding the mechanisms of change and informing personalized interventions. Moreover, existing studies have rarely examined how the trajectories of social support and loneliness unfold over time and interact at the individual level. This presents a significant gap in the literature, as loneliness is not a static condition but rather a dynamic and fluctuating experience (13). It is therefore necessary to adopt a longitudinal approach that allows for the modeling of intraindividual change and the temporal sequencing. Such an approach enables researchers to disentangle stable trait-level associations from state-level processes among older adults.

Loneliness is defined as a subjective emotional state characterized by feelings of alienation and indifference, which arise from a discrepancy between an individual’s desired social relationships and their actual social experiences (14). It reflects a state in which an individual’s need for emotional connection is unmet during social interactions (15). Extensive research has shown that loneliness has significant and lasting negative effects on the physical and mental health of older adults (4, 16). On the one hand, loneliness not only increases the risk of depressive symptoms and anxiety disorders but also reduces mental resilience and emotional stability (17, 18). On the other hand, loneliness can impair cognitive function, exacerbate chronic diseases, and even contribute to higher mortality rates (19, 20). Additionally, loneliness is dynamic and its severity tends to diminish over time (21). Given the well-documented adverse effects of loneliness on both the physical and mental health of older adults—including increased risks of depression, anxiety, cognitive decline, chronic disease exacerbation, and mortality (4, 16–20)—understanding its developmental course and modifiable predictors is of urgent practical importance. By employing a longitudinal design, the present study aims to (1) investigate whether changes in perceived social support are significantly associated with changes in loneliness over time at the within-person level, and (2) explore the temporal dynamics and bidirectional processes between these two constructs. This approach not only addresses a critical gap in the literature but also provides a more nuanced understanding of the social determinants of loneliness in aging populations, thereby informing targeted intervention strategies.

Based on Maslow’s hierarchy of needs, an individual’s mental health depends on the satisfaction of basic psychological needs, including belongingness needs, autonomy needs, and competence needs (22). Specifically, social support plays a crucial role in alleviating loneliness among older adults by addressing their core psychological needs, including belongingness, autonomy, and competence (23). That is, belongingness needs refer to the desire for stable and meaningful social relationships, which are often threatened in later life due to role transitions or social losses. Social support fulfills these needs by fostering emotional connection and providing a sense of acceptance, thereby reducing feelings of isolation (24). Autonomy needs, defined as the desire to maintain control over one’s life and decisions, are often undermined in older adults due to physical decline or dependency (25). Thus, it respects and reinforces their autonomy and enhances their sense of agency, thereby mitigating loneliness (26). Competence needs can also diminish with aging as the older adults experience reduced opportunities to contribute or demonstrate skills. Social support, particularly through activities that acknowledge and utilize their abilities, helps restore a sense of self-efficacy and purpose (27). By simultaneously addressing these interconnected needs, social support becomes a powerful mechanism to reduce loneliness and improve the psychological well-being of the older adults (28).

Besides, according to the attachment theory, which posits that people are physiologically predisposed to form strong emotional bonds with significant others to ensure security and emotional regulation (29). Although attachment behaviors are most prominent in early childhood, contemporary attachment research emphasizes that attachment needs persist throughout a person’s life (30). Among older adults, key life transitions-such as retirement and declining health-can disrupt established attachment relationships, leading to greater vulnerability to emotional distress, including loneliness. From this perspective, loneliness is conceptualized not just as a lack of social contact, but as a disruption of emotionally secure attachment bonds. Importantly, perceived social support can serve as a proxy for secure attachment. This means that from an attachment perspective, the quality of social support-not just its availability-is most important in addressing loneliness (31).

Empirical studies have indicated that the older adults with high social support show lower depression in cross-sectional study (11, 12, 31, 32). One longitudinal study also indicates that social support significantly negatively predicts loneliness of the older adults (13). In addition, a longitudinal study of 10,146 older adults (aged 70+) from the ASPREE cohort demonstrated that those who actively engage in community activities and maintain broad interpersonal networks tend to sustain more positive mental states, even as physical mobility declines (33). Also, a meta-analysis further confirms that social support is an important predictor of loneliness of the older adults (2). However, previous studies have three larger problems that weaken the validity of their findings. First, previous studies are mainly conducted in Western countries under the background of individualism. Second, they used a cross-sectional design, which raised the concern of shared method variance. Third, the existing literature remains unclear whether high social support can reduce loneliness and loneliness can affect the quality of social support at the within-person level. Thus, the present study was to explore the relation between social support and loneliness used a longitudinal design and the parallel process latent growth curve model among Chinese older adults.

It is important to note that, loneliness may also influence social support of the older adults according to Interactive process theory which emphasizes that social support is the product of a two-way interaction in which individuals are both recipients and providers of support (34). That is, when loneliness is excessive, older adults may exhibit negative interaction patterns, such as overdependence, emotional detachment, or distrust of others’ help, which can significantly affect their social support. Specifically, feelings of loneliness are usually accompanied by negative self-perceptions and distrust of the intentions of others. When older adults feel lonely, they may perceive themselves as unworthy of support or become skeptical of the concerns of others (21). This cognitive bias can prevent them from actively seeking social support while causing them to misinterpret the supportive behaviors offered by others (35). Namely, loneliness and social support may reciprocally affect each other. However, to date, none of the aforementioned studies has examined the possibility of bidirectional associations in the relation between loneliness and social support.

By incorporating the Maslow’s hierarchy of needs and the socio-emotional selectivity theory, we used a longitudinal design to address the relation between social support and loneliness by using cross-lagged model and the parallel process latent growth curve model. Based on the aforementioned literature, the aims of the current study were two fold. The first aim was to explore whether social support and loneliness were pairwise bidirectional related over time (Hypothesis 1). Specifically, older adults with high social support would be more likely to show low loneliness. Likewise, once older adults have high loneliness, they are more engaged in low social support. The second aim was to examine whether the intercept and slope of social support would be significantly related to the intercept and the slope of loneliness (Hypothesis 2). Specifically, the initial level of social support would significantly predict the initial level of loneliness and the change rate of social support would significantly predict the change rate of loneliness.

## Method

### Participants and sample

In the current study, six large communities in Wuhan, each with an approximate population of 30,000 and a history of around 20 years, were selected as the target population. A simple random sampling method was employed, supported by community committees and promotional activities in community squares to facilitate participant recruitment face-to-face surveys. Additionally, to increase the diversity and reach of the sample, a snowball sampling approach was utilized, whereby participants were encouraged to invite their friends and acquaintances to join the survey. The inclusion criteria specified that participants must be permanent residents of Wuhan with local household registration, aged 60 years or older, free from hearing or speech impairments, and without major health conditions such as dementia. Snowball sampling was deemed necessary due to the limitations of random recruitment in capturing certain segments of the older adult population, particularly those who were more socially isolated or less likely to engage with community activities.

**Figure 1:**
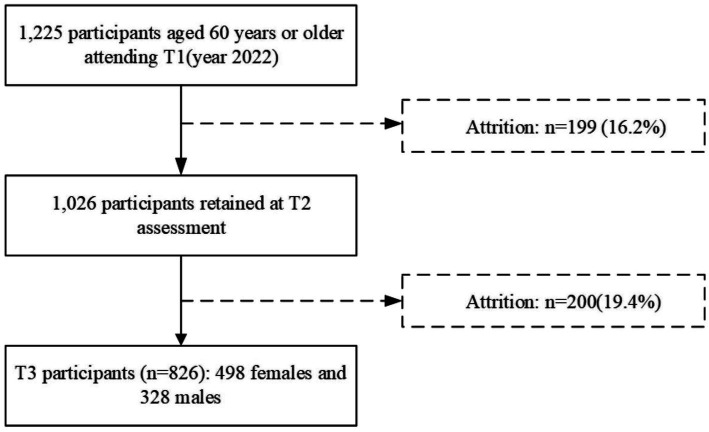
Flowchart of participants.

The study began by calculating the required sample size using G*Powe 3.1 software. To achieve 80% statistical test power, a significant level of α = 0.05 (two-tailed), and a medium effect size (*d* = 0.4), a minimum of 352 subjects was required. The survey was initiated in March 2022, with follow-up assessments conducted every 6 months thereafter. Using three waves of data collection on loneliness and social support among older adults, a cross-lagged model and a parallel latent variable growth model were constructed. The initial wave (T1) yielded 1,225 valid responses. The second wave (T2) collected 1,026 valid responses, and the third wave (T3) obtained 826 valid responses, comprising 328 males and 498 females, after excluding participants who were lost to follow-up or declined further participation. Given the critical role of sample size in longitudinal studies, this research also analyzed sample attrition rates. Specifically, the attrition rate for the first wave was 16.2%, while the attrition rate for the second wave was 19.4% (Figure 1).

### Data collection

This longitudinal study involved face-to-face data collection conducted by trained undergraduate students in educational psychology and provided detailed explanations to older adults and their families regarding the research purpose, procedures, and potential risks, followed by the distribution of informed consent forms. Participants were formally enrolled only after providing consent. During the intervals between assessments, regular contact was maintained with participants through phone follow-ups and community meetings to check on their well-being and remind them of the timing and importance of the next assessment. For participants at risk of dropping out due to health issues, relocation, or other reasons, accommodations (such as home visits or assistance in resolving practical difficulties) were provided whenever possible to enhance retention rates and ensure data continuity and completeness. Questionnaires were collected immediately after each assessment. For participants who had difficulty reading, writing, or understanding questionnaire items, the trained interviewers offered one-on-one assistance, including reading questions aloud, clarifying their meaning using Mandarin or local dialects, and accurately recording the participants’ responses without interpretation or leading. Given the longitudinal design of this study, the three assessments were conducted at six-month intervals, with consistent testing procedures each time. The total duration of each assessment was approximately 30 min.

### Assessment of social support

The Navigating the Social Support Scale was used to measure levels of social support (36). The questions of the scale were set around the three dimensions of family, friends and other support, with a total of 12 questions. A 7-point Likert scale (1 strongly disagree to 7 strongly agree) was used. Higher scores indicate a greater sense of social support felt by the individual. The Cronbach’s α were 0.94, 0.95, and 0.93.

### Assessment of loneliness

We used the third version of the UCLA Loneliness Scale developed by Russell (37). This version reduces the reading skills required to comprehend the entries and is well suited to the older population. The scale consists of 20 questions, of which 11 are in positive order and 9 are in reverse order (which have been converted to positive order). The scoring system is based on a four-point scale, with higher scores obtained indicating a greater degree of loneliness in the individual. The Cronbach’s α were 0.95, 0.95, and 0.95.

### Data analysis

First, descriptive statistics and correlation analyses were performed using SPSS 26.0 as an initial step in the data analysis. Second, structural equation modeling was conducted in Mplus 8.3 to address the primary research questions. Specifically, the measurement invariance of all scales over time was assessed, and the relative fit of the constrained model was evaluated based on changes in CFI and RMSEA. The changes in CFI and RMSEA for each model were less than 0.010 and 0.015, respectively, indicating that the models demonstrated equivalent fit (38). Additionally, a three-wave autoregressive cross-lagged panel model (CLPM) was constructed to examine the cross-lagged effects and bidirectional associations between social support and loneliness. Finally, a parallel process latent growth curve model was employed to investigate the longitudinal relationship between social support and loneliness over time. Several model fit criteria were employed to evaluate goodness-of-fit (39), which included the chi-square/degrees of freedom ratio (*χ*^2^/*df*), the comparative fit index (CFI), the Tucker and Lewis Index (TLI), the root mean squared error of approximation (RMSEA), and the standardized root mean square residual (SRMR). Close fit of the model was indicated by *χ*^2^/*df* values ≤ 3 (39), TLI and CFI values ≥ 0.90 (40), RMSEA values ≤ 0.08 (41), and SRMR values ≤ 0.08 (42).

## Results

### Bivariate analyses

Means, standard deviations, and correlations for all variables are presented in Table 1. Social support was negatively associated with loneliness at all times.

**Table 1:** Descriptive statistics and correlations among variables of interest.

	*M*	*SD*	1	2	3	4	5	6
1. T1 loneliness	35.03	13.93	1					
2. T2 loneliness	38.12	14.55	0.529^***^	1				
3. T3 loneliness	41.34	14.86	0.388^***^	0.540^***^	1			
4. T1 social support	54.49	19.38	−0.116^***^	−0.200^***^	−0.230^***^	1		
5. T2 social support	52.24	19.58	−0.197^***^	−0.368^***^	−0.235^***^	0.562^***^	1	
6. T3 social support	52.13	18.68	−0.279^***^	−0.360^***^	−0.470^***^	0.476^***^	0.545^***^	1

^*^p < 0.05, ^**^p < 0.01, ^***^p < 0.001.

### Testing for the cross-lagged regression of social support and loneliness

First, to assess the measurement invariance of all scales over time, we established the morphological equivalence model, weak equivalence model, and strong equivalence model for the two constructs of social support and loneliness after control socioeconomic. The model fit indices are presented in Table 2. Following the recommendation of Cheung and Rensvold (43), ΔCFI was used as a criterion for model comparison, as it is not influenced by model parameters or sample size. When ΔCFI is equal to or less than 0.01, the assumption of measurement invariance is supported. This confirmation allowed for subsequent cross-lagged analyses to be conducted.

**Table 2:** Cross-temporal equivalence test for loneliness and social support.

Measurement model	*χ*^2^ (*df*)	ΔCFI	ΔTLI	CFI	TLI	RMSEA	(90% CI)	SRMR
Social support								
Morphological equivalence	698.372 (510)	–	–	0.923	0.916	0.058	(0.043, 0.064)	0.063
Weak equivalence	752.712 (538)	−0.009	−0.006	0.914	0.910	0.055	(0.043, 0.065)	0.079
Strong equivalence	812.456 (543)	−0.008	−0.005	0.906	0.905	0.053	(0.045, 0.065)	0.089
Loneliness								
Morphological equivalence	363.452 (148)	–	–	0.991	0.988	0.040	(0.037, 0.047)	0.018
Weak equivalence	372.523 (169)	0.001	0.003	0.992	0.991	0.037	(0.035, 0.043)	0.019
Strong equivalence	392.682 (192)	0.002	0.002	0.994	0.991	0.039	(0.033, 0.042)	0.019

**Figure 2:**
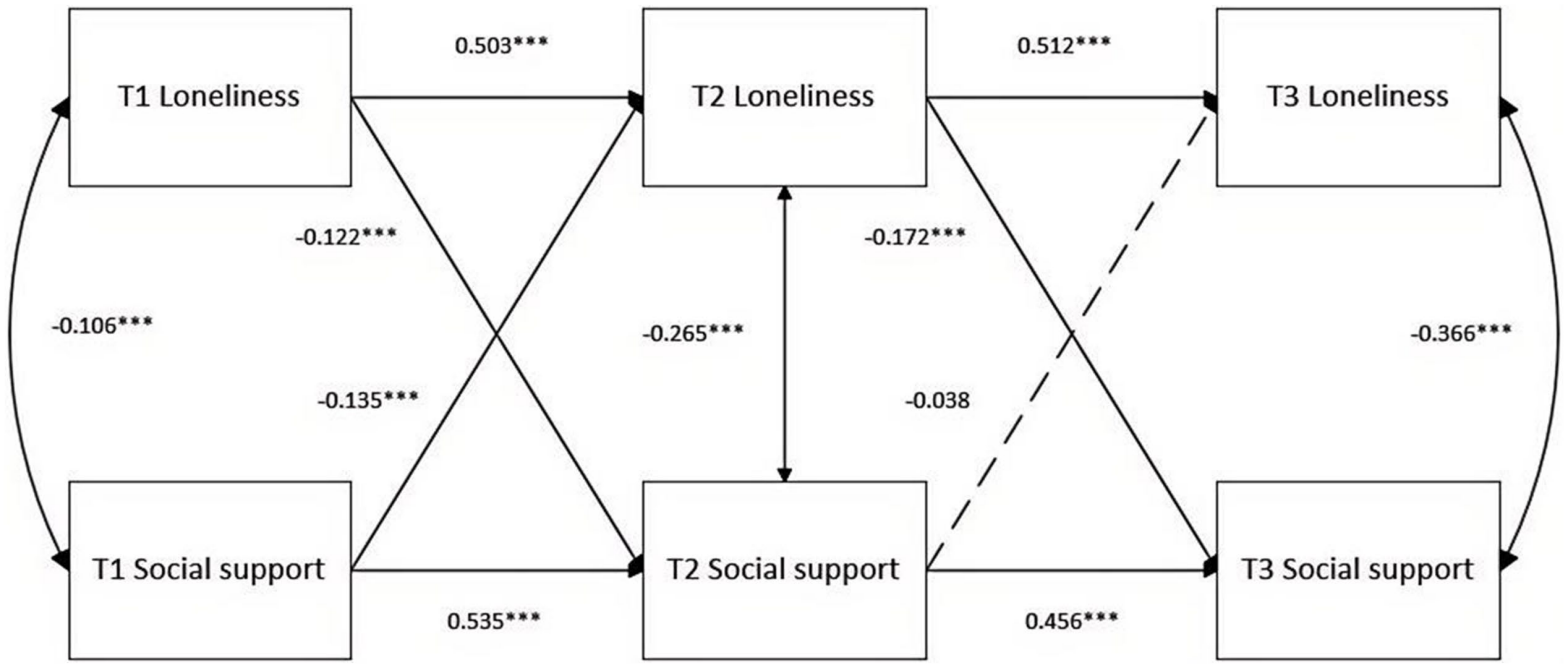
Cross-lagged model with longitudinal relations between social support and loneliness. The symbol * denotes statistical significance levels. *p < 0.05, **p < 0.01, ***p < 0.001.

Second, after control socioeconomic, the model was a saturated model with *χ*^2^ (32) = 0, RMSEA = 0, CFI = 1, and TLI = 1. The results are shown in Figure 2. The autoregressive path analysis results with the same variables at different time points showed that the loneliness and social support showed moderate stability at three-time points, with the autoregressive path coefficients ranging from 0.456 to 0.535. More importantly, the cross-regression paths analysis showed that loneliness at Time 1 had significant predictive effects on social support at Time 2 (β = −0.122, *p* < 0.001). Social support at Time 1 had significant predictive effects on loneliness at Time 2 (β = −0.135, *p* < 0.001). Loneliness at Time 2 had significant predictive effects on social support at Time 3 (β = −0.172, *p* < 0.001). Social support at Time 2 could not significantly predict loneliness at Time 3.

### Parallel latent variable growth modeling of social support and loneliness

**Figure 3:**
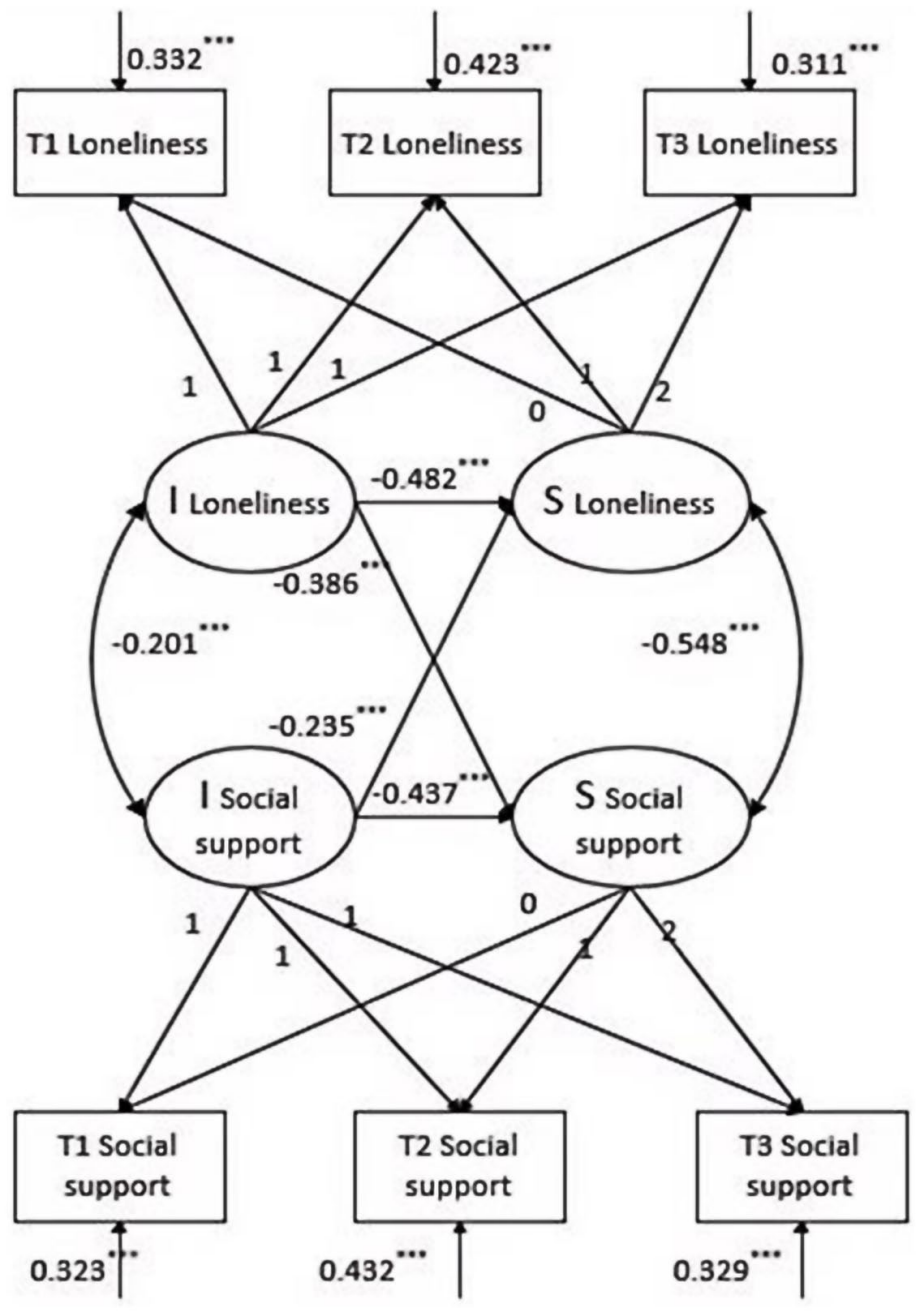
The parallel process latent growth curve model between social support and loneliness. The symbol * denotes statistical significance levels. *p < 0.05, **p < 0.01, ***p < 0.001.

As shown in Figure 3, after control socioeconomic, the model fit well, *χ*^2^/*df* = 9.892, RMSEA = 0.102, SRMR = 0.038, CFI = 0.957, and TLI = 0.911. Loneliness was negatively correlated with social support at the initial level (*r* = −0.201, *p* < 0.001), i.e., the higher the initial loneliness of an individual, the lower the level of his or her social support. In addition, the rate of development of loneliness was negatively correlated with the rate of development of social support (*r* = −0.548, *p* < 0.001), which means that the faster an individual’s loneliness develops, the slower the level of social support develops. The initial level of loneliness negatively predicted the rate of development of social support level (β = −0.386, *p* < 0.01), which means that the higher the initial loneliness of an individual, the slower the rate of development of social support level, which acted as a hindrance. At the same time, the initial level of social support negatively predicted the rate of change in the level of loneliness (β = −0.235, *p* < 0.001), i.e., the higher the initial level of social support, the slower the rate of growth of loneliness, showing a buffering effect.

## Discussion

### Loneliness and social support

The current study employed cross-lagged regression modeling to investigate the dynamic relation between loneliness and social support. First, the research findings reveal a dynamic reciprocal relationship between loneliness and social support among older adults, which aligns with previous research conclusions (44). Specifically, higher baseline loneliness scores predicted lower subsequent social support levels. This phenomenon can be explained by the scar theory (45), which posits that negative emotional experiences leave lasting “scars” that impair individuals’ social functioning and foster pessimistic social expectations. In other words, compared to older adults with lower loneliness, those with higher loneliness tend to exhibit more negative social attitudes. The lonelier individuals were initially, the more pessimistic they became about future social interactions and self-perceptions (46). Thus, elevated loneliness in older adults may adversely affect their later social support.

Second, we found that higher levels of social support in the first year significantly reduced loneliness in the subsequent year (11, 12, 31). Our study also supports Maslow’s hierarchy of needs, which provides insight into how social support affects older adult’s loneliness from the perspective of psychological needs. The need for belonging gives older persons a sense of social connection, the need for autonomy gives them a sense of control over their lives, and the need for competence helps them to recognize their own value. The fulfillment of these needs makes social support an important psychological resource for alleviating loneliness among older adults (22). But, we found that social support in the second year did not significantly predict loneliness in the third year. This can be explained that, the alleviation of loneliness depends on the balance between the benefits and costs of social interactions. When older adults perceive the benefits of social support, such as emotional satisfaction or practical assistance, as exceeding the associated costs, such as time and energy investment, their loneliness decreases (32, 47). Conversely, if the perceived quality of support diminishes, such as through superficial family care or unengaging community activities, and the returns fall short of expectations, loneliness may intensify. These findings suggest that the effectiveness of social support in mitigating loneliness is influenced by variations in the quality of the support provided, highlighting an important direction for future research.

Third, the analysis of the latent variable growth model revealed a nuanced interaction between the initial state and developmental trends in the relationship between loneliness and social support among older adults. At the initial level, a significant negative correlation was identified: individuals with higher levels of loneliness perceive lower levels of social support (2). The reason is that, for the older adults, this effect may become more pronounced as a result of significant events such as changes in life roles (e.g., retirement), adjustments in life circumstances or relationship networks, or the loss of an intimate partner (13). What’s more, the study also revealed that older adults with higher initial levels of social support exhibited slower growth in loneliness over time, even though their social support levels may also decline. This can be explained that, higher initial social support reflects greater access to social capital, which provides emotional comfort and a sense of belonging over time (1, 48).

Fourth, a notable finding of this study was the relationship between initial levels of loneliness and subsequent developmental trajectories. Older adults with higher initial levels of loneliness exhibited slower increases in loneliness over time but also experienced slower progress in social support. This seemingly paradoxical finding reflects the complexity of psychological adaptation mechanisms in later life (49). Higher initial loneliness may indicate that individuals have developed stable coping strategies, which mitigate their perception of loneliness (50, 51). However, these adaptations are often accompanied by a narrowing of social networks, leading to a stable but negative equilibrium (52). This phenomenon underscores a distinctive pattern of emotional regulation and social interaction among older adults and highlights the bidirectional relationship between loneliness and social support. The findings have practical implications for designing mental health interventions, emphasizing the importance of rebuilding social support systems for individuals with high levels of loneliness to disrupt cycles of negative adaptation (53).

Given that this study was conducted only in China, it is necessary to consider how the socio-cultural context influences the manifestation of loneliness and the nature of perceived social support. In Chinese society, where Confucian values are deeply rooted, family-based collectivism and filial piety remain central to intergenerational relationships and care arrangements for older people. Social support usually comes primarily from close relatives, especially adult children, and cultural expectations of interdependence may exacerbate the psychological impact of unmet support needs. Thus, there is a negative correlation between loneliness and subsequent social support, and initial levels of support are also protective. In contrast, studies from Western individualistic societies typically report a wider range of sources of support for older people, including neighbors and community-based organizations formal social services. These differences reflect different cultural patterns of aging and self-identity, that is, East Asian societies emphasize interdependence and role-based obligations, while Western societies tend to value autonomy and voluntary association. For example, in the West, older adults may have greater flexibility to reconfigure their social networks and seek emotional fulfillment outside the home, potentially buffering the long-term effects of loneliness. Thus, the mechanisms by which social support influences loneliness, and the reciprocity of such relationships over time may vary by culture.

### Limitations and implications for interventions

Several limitations and directions for future research should be considered to provide a balanced interpretation of the findings. First, all measures relied on self-reports from older adults, which may introduce biases and affect the validity of the results. Future studies should aim to collect data from multiple sources to enhance objectivity. Besides, we acknowledge that a snowball sampling method may introduce potential selection bias, as referred individuals may share similar characteristics with initial participants. Second, the current study employed a six-month interval between measurements. Future research could benefit from extending the study period and increasing the frequency of data collection to capture more detailed developmental trajectories. Lastly, the sample primarily consisted of older adults from China, which may restrict the generalizability of the findings. Future studies should examine these relationships in diverse cultural contexts to better understand their universality and cultural specificity.

However, despite these limitations, the findings of the current study offer meaningful implications for both educational and psychological practice. First, previous cross-sectional designs have been limited in their ability to accurately predict the directionality and dynamic changes in the relationship between loneliness and social support. To address this limitation, the present study employs a cross-lagged model and a parallel latent variable growth model, enabling a more comprehensive exploration of the interaction patterns and developmental trajectories between the two constructs from a dynamic perspective. This approach provides robust empirical evidence to deepen understanding. Second, the study carries important practical implications. Given that social support is a critical factor in alleviating loneliness, the findings underscore the need for older adults service organizations, community workers, and families to actively expand and strengthen social support systems for older adults, offering actionable guidance for interventions and policy development. That is, efforts should be made to foster meaningful interpersonal connections, such as through peer-support groups, intergenerational programs, or community-based participatory activities that enhance agency, recognition, and relational satisfaction. Third, family support can be strengthened through intergenerational bonding programs like “Grandparent-Grandchild Connection” and educational initiatives. Providing free mental health services, including counseling and hotlines, is essential to address psychological needs. Bridging the digital divide through training programs and user-friendly social platforms can help older adults connect more easily. Thus, a lifespan approach to social engagement should be encouraged in policy, wherein participation in civic organizations, volunteering, and social networks is promoted well before old age. Finally, public awareness campaigns should emphasize the importance of older adults mental health and encourage community-wide involvement in support efforts. From a public health perspective, this suggests that loneliness should not merely be treated as an outcome, but also as a precursor to future social withdrawal. Routine screening for loneliness, integrated into community health services and geriatric assessments, may facilitate timely referrals and personalized psychosocial interventions.

## Conclusion

The current study examined the relation between social support and loneliness used a longitudinal design and the parallel process latent growth curve model among Chinese older adults. Our findings highlight that loneliness of the older adults increases over time, while social support slowly decreases. Moreover, initial levels of loneliness negatively predicted the rate of development of social support, and social supports also negatively predicted the rate of development of loneliness. These results emphasize the importance of measures to effectively alleviate the sense of isolation of the older adults, enhance their level of social support and promote mental health and quality of life.

## Data Availability

The raw data supporting the conclusions of this article will be made available by the authors, without undue reservation.
